# Explaining variance in self-directed learning readiness of first year students in health professional programs

**DOI:** 10.1186/s12909-017-1043-8

**Published:** 2017-11-13

**Authors:** Craig E. Slater, Anne Cusick, Jimmy C. Y. Louie

**Affiliations:** 1 School of Health and Society, University of Wollongong Australia, Wollongong, NSW 2522 Australia; 2 College of Health and Rehabilitation Sciences: Sargent College, Boston University, Boston, USA; 3 Faculty of Health Sciences, University of Sydney, Lidcombe, NSW 2141 Australia; 4 School of Biological Sciences, The University of Hong Kong, Pokfulam, Hong Kong

**Keywords:** Self-directed learning, Education, Professional, Interdisciplinary studies, Students, Health occupations

## Abstract

**Background:**

Self-directed learning (SDL) is expected of health science graduates; it is thus a learning outcome in many pre-certification programs. Previous research identified age, gender, discipline and prior education as associated with variations in students’ self-directed learning readiness (SDLR). Studies in other fields also propose personality as influential.

**Method:**

This study investigated relationships between SDLR and age, gender, discipline, previous education, and personality traits. The Self-Directed Learning Readiness Scale and the 50-item ‘big five’ personality trait inventory were administered to 584 first-year undergraduate students (*n* = 312 female) enrolled in a first-session undergraduate interprofessional health sciences subject.

**Results:**

Students were from health promotion, health services management, therapeutic recreation, sports and exercise science, occupational therapy, physiotherapy, and podiatry. Four hundred and seven responses (*n* = 230 females) were complete. SDLR was significantly higher in females and students in occupational therapy and physiotherapy. SDLR increased with age and higher levels of previous education. It was also significantly associated with ‘big five’ personality trait scores. Regression analysis revealed 52.9% of variance was accounted for by personality factors, discipline and prior experience of tertiary education.

**Conclusion:**

Demographic, discipline and personality factors are associated with SDLR in the first year of study. Teachers need to be alert to individual student variation in SDLR.

## Background

Universities and academic programs have long had interest in understanding students’ readiness to engage in self-directed learning (SDL). Understanding pre-certification students’ readiness for SDL can assist educators to meaningfully and effectively prepare students for SDL in a professional and practice context. The classic definition by Knowles [1] describes SDL as “a process in which individuals take the initiative, with or without the help of others, in diagnosing their learning needs, formulating learning goals, identifying human and material resources for learning, choosing and implementing appropriate learning strategies, and evaluating learning outcomes” (p.18). While SDL is a desired academic and professional trait, there is little insight into what might make students ‘ready’ to engage in this process. In the case of pre-certification students, will they be ‘ready’ for SDL as a result of attributes and experience they bring with them to training? Alternatively, will they be ‘ready’ as a result of their teaching and learning experiences?

As such, student self-directed learning readiness (SDLR) has been the subject of research inquiry. A recent review of the literature relating to factors associated with students’ SDLR revealed that demographic, educational, and discipline and factors were most commonly investigated [2].

Increasing age has been consistently associated with increasing levels of SDLR [3–8]. Previous levels of education also positively affect SDLR; that is, the higher the qualification on entry into the program, the higher the SDLR [3, 9]. It takes time to get higher qualification levels, so this could be a function of age, but this has not been independently assessed. The time/age interaction could also explain why year levels show differences. A number of studies show SDLR increases with program progression [10–13]; although equally this could relate to the teaching and learning experiences in the programs. This, however, has generally not been teased out. The fact that some studies [4, 14, 15] have found SDLR *decreases* with increasing program level is illustrative. Perhaps teaching and learning experiences over time rather than age per se are more influential.

Gender is the other factor most commonly investigated in pre-certification health professional program cohorts [2]. The relationship between gender and SDLR was significant in only two studies [4, 16] which had opposite results. In many studies, the association between gender and SDLR had not been meaningfully explored because samples have been overwhelmingly female. This may in part account for the ambiguity about association.

Discipline has received limited research attention. Most studies use single-discipline samples: medicine [3, 8, 16, 17]; nursing [9, 13, 18, 19]; physiotherapy [4, 5, 20, 21]; pharmacy [22–24]; and dentistry [15]. Few studies use multidisciplinary cohorts, and of those that do, comparison between disciplines has not been of particular interest. Linares [7] and Malta et al. [25] found differences in SDLR between select programs, however, in both studies these differences were not statistically investigated. No studies to date have included cohorts in specifically interprofessional classes.

Apart from age, gender, prior education level and discipline, quantitative investigation into other educational, program and academic determinants of SDLR levels has been scant. Surprisingly, there are few studies exploring the influence of traits, such as personality, which has shown influence on student academic success in general [26]. This is in spite of an emerging debate in literature regarding whether or not SDLR can actually be ‘taught’. Lounsbury et al. [27], for example, suggests that SDLR is in fact an attribute or trait, rather than a self-report proxy for performance capacity which can change over time. Surprisingly, although standardised tools have been available for many decades to investigate individual traits such as personality, to date no research has explored whether traits such as personality are associated with SDLR levels in health disciplines in general and pre-certification programs in particular.

This study examined factors previously found to be associated with SDLR level – gender, age, previous educational level and discipline – and included a measure of personality as a new factor. The study then considered whether the factors of most influence were amenable to instruction. The study builds on the evidence base by investigating previously explored factors in a new cohort; first year undergraduate pre-certification students engaged in interprofessional study at the beginning of their program. Some of the disciplines in this cohort have never previously had SDLR data presented. The study presents SDLR profiles for the cohort as a whole and for each discipline. It investigated associations for the cohort as a whole and for each discipline; and for the whole cohort a regression model is presented.

## Method

A single cohort cross-sectional survey design was used. Ethics approvals were obtained from the Western Sydney University Human Research Ethics Committee (H9857) and the University of Wollongong/Illawarra and Shoalhaven Local Health District Human Research Ethics Committee (HE12/226) including approval to extract student demographic and program data from institutional records, and match to completed surveys. The matched dataset was de-identified for research purposes.

### Participants

A total of 584 undergraduate students were enrolled in in a first session, first year interprofessional health science subject at a metropolitan university in New South Wales, Australia. Enrolled students were from the following programs: health sciences (majors in therapeutic recreation, health promotion and health services management) (*n* = 158); sport and exercise science (*n* = 215); occupational therapy (*n* = 86); physiotherapy (*n* = 71); and podiatry (*n* = 54).

### Instruments

Students completed the Self-Directed Learning Readiness Scale (SDLRS) [28] and the ‘big five’ personality trait inventory from the International Personality Item Pool (IPIP) [29].

The SDLRS is a 58 item self-report survey measuring the complex of attitudes, skills, and characteristics that comprise an individual’s current level of readiness to manage his or her own learning. The SDLRS is the most commonly used instrument to measure self-directed learning readiness in pre-certification cohorts in the health disciplines [2]. The instrument asks participants to select one of five responses which best reflects their own attitude or preference towards learning. Total scores range from 58 to a possible total of 290, with higher scores indicating increased readiness for self-directed learning. Guglielmino and Associates [30] reported a mean score of 214 ± 25.6 within a range of 189 to 240 in the general adult population. Mean scores of undergraduate students in health disciplines have ranged between 208.48 ± 17.62 [11] and 238.70 ± 21.0 [3]. The instrument has a split-half reliability of *r* = 0.94 [31] and test-retest reliability coefficients of *r* = 0.79 [32] and *r* = 0.82 [33], as reported by Guglielmino and Associates. There is a nursing version called the Self-Directed Learning Readiness Scale for Nursing Education (SDLRSNE) [34]. The SDLR was selected for the study because there were no nurses in this sample.

The ‘big five’ personality trait inventory is a 50-item inventory derived from Goldberg’s [35] 100-item inventory and is commonly used in the interest of time efficiency. There are ten items related to each of the five factors: ‘extroversion’, ‘agreeableness’, ‘conscientiousness’, ‘emotional stability’, and ‘intellect/imagination’. Participants choose one of five responses which reflect how accurately the item describes them. Responses to items are scored between one and five, and the sum is tallied for each of the five factors. Total scores range from a minimum of 10 to possible total of 50 for each factor. The instrument has a mean internal consistency across the five factors of α = 0.84, and average correlation with factor markers of *r* = 0.67 and as per the IPIP website (http://ipip.ori.org/). Further work by Gow et al. [36] showed internal consistencies ranging α = 0.72 to α = 0.87 in a university student population in Scotland and confirmed the five-factor structure.

### Data collection

All students were asked to complete the surveys online within the first 12 weeks of program commencement as a non-assessable learning activity. Students could give consent for responses to be used in research. Permission was granted by the university for the following data to be extracted from enrolment records and linked to survey responses: age, gender, program of enrolment, and highest level of previous education. All matched data were de-identified by an independent administrative officer prior to researcher access.

### Data analysis

Data were analysed using SPSS version 22.0 (IBM Corp., Armonk, NY, USA). Descriptive statistics were used to characterise the sample on demographic, academic, personality and SDLRS variables. One-way analysis of variance (ANOVA) were used to compare SDLRS scores between academic programs and students’ educational backgrounds. *Post-hoc* Bonferroni analyses were then conducted to determine significant differences between specific academic programs and educational backgrounds. An independent t-test was used to compare mean SDLRS scores of males and females. Pearson’s correlation was used to identify relationships between SDLRS scores and both age and personality factors. Effect size of the potential determinants on SDLRS scores was calculated using Cohen’s *d* for t-test analyses, and eta squared (η^2^) for ANOVA analyses. Multiple regression was conducted to construct a model explaining the relationship between investigated factors and SDLRS scores. Statistical significance was set at *p* < 0.05.

## Results

### Characteristics of the sample

The survey was completed by 456 students, a response rate of 78%. Of these, 407 consented to have their responses used in the research. Demographic attributes and mean academic results of the sample across programs are presented in Table 1. Overall, there were more females than males and across programs (177 male, 230 female) which was statically significant, *χ* ^2^(4) = 78.33, *p* < 0.001. Most students had entered the program following completion of their final year of secondary education. Across programs, there was also a significant difference between student’s highest level of previous education, *χ* ^2^(24) = 50.86, *p* < 0.001. Podiatry had greater representation of students who had an incomplete or complete bachelor’s degree, and fewer students who had completed secondary education than the expected counts. There were no significant differences across programs in the personality factors ‘extroversion’, ‘emotional stability’ or ‘intellect/imagination’. There were, however, differences in the factors ‘agreeableness’ *F*(4398) = 5.45, *p* < 0.001, and ‘conscientiousness’ *F*(4398) = 3.02, *p* = 0.02. The mean age across programs was 20 years, with differences in age across programs *F*(4402) = 5.250, *p* < 0.001.

**Table 1:** Characteristics across programs

	Health Science	Sports and Exercise Science	Occupational Therapy	Physiotherapy	Podiatry	TOTAL
*n (%)*	94 (23.1)	148 (36.4)	69 (17.0)	61 (15.0)	35 (8.6)	407
Gender, *n (%)*						
Male	22 (23.4)	95 (64.2)	7 (10.1)	32 (52.5)	21 (60.0)	177 (43.5)
Female	72 (76.6)	53(35.8)	62 (89.9)	29 (47.5)	14 (40.0)	230 (56.5)
Highest level of previous education, *n (%)*						
No previous qualification/other qualification	3 (3.2)	7 (4.7)	1 (1.4)	0 (0.0)	0 (0.0)	11 (2.7)
Complete secondary education	62 (66.0)	92 (62.2)	40 (58.0)	44 (72.1)	12 (34.3)	250 (61.4)
Incomplete vocational level program	1 (1.1)	4 (2.7)	0 (0.0)	0 (0.0)	0 (0.0)	5 (1.2)
Complete vocational level program	8 (8.5)	21 (14.2)	7 (10.1)	1 (1.6)	4 (11.4)	41 (10.1)
Incomplete bachelor’s degree level program	12 (12.8)	23 (15.5)	17 (24.6)	11 (18.0)	13 (37.1)	76 (18.7)
Complete bachelor’s degree level program	5 (5.3)	1 (0.7)	3 (4.3)	4(6.6)	5 (14.3)	18 (4.4)
Unknown	3 (3.2)	0 (0.0)	1 (1.4)	1 (1.6)	1 (2.9)	6 (1.5)
Personality factor, *mean ± SD*						
Extroversion	33.76 ± 6.42	35.20 ± 6.03	33.42 ± 6.74	33.90 ± 5.54	33.26 ± 7.30	34.21 ± 6.31
Agreeableness	41.86 ± 5.38^a^	39.49 ± 5.56^ab^	42.73 ± 5.41^b^	40.49 ± 4.57	40.14 ± 5.73	40.78 ± 5.49
Conscientiousness	35.08 ± 6.15	33.54 ± 6.15^c^	36.28 ± 6.38^c^	35.84 ± 6.63	35.51 ± 5.87	34.86 ± 6.30
Emotional stability	31.30 ± 8.79	33.66 ± 7.74	31.73 ± 7.16	33.46 ± 7.83	32.65 ± 8.44	32.69 ± 8.00
Intellect/imagination	36.21 ± 6.52	35.84 ± 5.03	36.46 ± 5.48	36.70 ± 5.86	34.71 ± 6.09	36.06 ± 5.69
Mean age, *years ± SD*	19.70 ± 3.44 ^d^	19.79 ± 4.49 ^e^	21.43 ± 6.72	19.21 ± 4.03 ^f^	23.17 ± 7.41^def^	20.25 ± 5.08

^a^Difference between sport and exercise science and health science students was statistically significant (*p* = 0.01)

^b^Difference between sport and exercise science and occupational therapy students was statistically significant (*p* = 0.001)

^c^Difference between sport and exercise science and occupational therapy students was statistically significant (*p* = 0.03)

^d^Difference between podiatry and health science students was statistically significant (*p* = 0.005)

^e^Difference between podiatry and sport and exercise science students was statistically significant (*p* = 0.003)

^f^Difference between podiatry and physiotherapy students was statistically significant (*p* = 0.002)

### Gender

Females had higher SDLR than males. As shown in Table 2, SDLRS scores were significantly higher for females (215.53 ± 25.46) than for males (209.11 ± 23.19), *t*(405) = 2.62, *p* = 0.009, *d* = 0.264. Both scores are within the range for average self-directed learning readiness (202–226) [30].

**Table 2:** SDLRS scores by program and demographic factors

	Health Science (*n* = 94)	Sports and Exercise Science (*n* = 148)	Occupational Therapy (*n* = 69)	Physiotherapy (*n* = 61)	Podiatry (*n* = 35)	TOTAL (*n* = 407)
	Mean ± SD	Mean ± SD	Mean ± SD	Mean ± SD	Mean ± SD	Mean ± SD
Cohort	209.47 ± 27.21*	207.51 ± 22.66^+^^	220.29 ± 24.86*^+^	219.23 ± 22.40^^^	217.40 ± 23.26	212.74 ± 24.68
Gender						
Male	202.14 ± 21.27	204.81 ± 21.06	214.86 ± 34.94	220.59 ± 23.12	216.43 ± 23.63	209.11 ± 23.19
Female	211.71 ± 28.54	212.36 ± 24.75	220.90 ± 23.77	217.72 ± 21.89	218.86 ± 23.51	215.53 ± 25.46
Highest level of previous education						
No previous qualification / other qualification	225.00 ± 9.17	210.43 ± 25.48	#	–	–	217.45 ± 22.98
Complete secondary education	202.21 ± 24.83	205.78 ± 22.81	217.93 ± 24.14	213.73 ± 20.92	210.25 ± 21.56	208.45 ± 23.67
Incomplete vocational level program	#	206.00 ± 20.15	–	–	–	204.40 ± 17.81
Complete vocational level program	223.63 ± 20.44	210.57 ± 17.65	223.71 ± 30.29	#	212.00 ± 34.24	215.98 ± 22.33
Incomplete Bachelor degree level program	223.75 ± 34.62	208.65 ± 24.53	221.88 ± 25.17	232.55 ± 23.70	222.62 ± 22.18	219.84 ± 26.61
Complete Bachelor degree level program	233.00 ± 27.27	#	220.33 ± 34.39	237.75 ± 16.15	226.80 ± 23.89	231.83 ± 24.24
Unknown	213.67 ± 22.50	–	#	#	#	220.17 ± 18.62

^#^Data not presented as only one respondent

*Difference between health science and occupational therapy was statistically significant (*p* = 0.05)

+Difference between sport and exercise science and occupational therapy students was statistically significant (p = 0.003)

^Difference between sport and exercise science and physiotherapy students was statistically significant (*p* = 0.016)

### Age

Older students had higher SDLR, than younger students. As shown in Table 3, there was a weak positive correlation between SDLRS scores and age (*r* = 0.266, *p* < 0.001).

**Table 3:** SDLRS score correlation with age and personality factor

	Health Science (*n* = 94)		Sports and Exercise Science (n = 148)		Occupational Therapy (n = 69)		Physiotherapy (n = 61)		Podiatry (n = 35)		TOTAL (n = 407)	
	*r*	*p*	*r*	*p*	*r*	*p*	*r*	*p*	*r*	*p*	*r*	*p*
Age	0.390	< .001	0.208	0.011	0.159	0.193	0.349	0.006	0.368	0.030	0.266	< 0.001
Personality factor												
Extroversion	0.224	0.032	0.292	< 0.001	0.191	0.121	0.156	0.229	0.444	0.008	0.222	< 0.001
Agreeableness	0.331	0.001	0.407	< 0.001	0.582	< 0.001	0.439	< 0.001	0.587	< 0.001	0.441	< 0.001
Conscientiousness	0.490	< 0.001	0.406	< 0.001	0.377	0.002	0.565	< 0.001	0.610	< 0.001	0.477	< 0.001
Emotional stability	0.291	0.005	−0.009	0.910	0.231	0.060	0.169	0.193	0.545	0.001	0.172	0.001
Intellect /imagination	0.616	< 0.001	0.506	< 0.001	0.620	< .001	0.486	< 0.001	0.477	0.004	0.541	< 0.001

### Highest level of previous education

SDLRS scores differed significantly depending on the student’s highest level of previous education *F*(6400) = 4.720, *p* < 0.001, η^2^ = 0.066. A post-hoc Bonferonni test revealed that there was a statistically significant difference between students who had completed their final year of secondary education (208.45 ± 23.67) and students who had previously commenced a bachelor level program but not completed (219.84 ± 26.61; *p* = 0.005); and students who had completed their final year of secondary education and students who had previously completed a bachelor level degree (231.83 ± 24.24; *p* = 0.001).

### Disciplinary differences

There was a statistically significant difference in mean SDLRS scores across disciplines *F*(4402) = 5.267, *p* < 0.001, η^2^ = 0.05 (Table 2). A post-hoc Bonferroni test showed that the difference in mean SDLRS scores was statistically significant between students in (a) health sciences (therapeutic recreation, health promotion and health services management) (209.47 ± 27.21) and occupational therapy (220.29 ± 24.86; *p* = 0.05); (b) sports and exercise science (207.51 ± 22.66) and occupational therapy (220.29 ± 24.86; *p* = 0.003); and (c) sports and exercise science (207.51 ± 22.66) and physiotherapy (219.23 ± 22.40; *p* = 0.016).

### Personality

SDLR was associated with increased scores on each of the ‘big five’ personality factors. In the whole cohort, there was a weak positive correlation with SDLR and both ‘emotional stability’ (*r* = 0.17, *p* = 0.001) and ‘extroversion’ (*r* = 0.22, *p* < 0.001). There was a moderate positive correlation with ‘agreeableness’ (*r* = 0.44, *p* < 0.001) and ‘conscientiousness’ (*r* = 0.48, *p* < 0.001). There was a strong positive correlation with ‘intellect/imagination’ (*r* = 0.541, *p* < 0.001).

### Accounting for variability in SDLR

Multiple regression analysis was used to develop a model for explaining variance in SDLR from the factors investigated: age, gender, previous level of education and personality. Descriptive statistics and regression coefficients are presented in Table 4. Due to insufficient numbers, students whose previous educational background was unknown (*n* = 6) and students who had no previous/other educational background (*n* = 11) were not included in the regression. Educational background was grouped as 0 = completed secondary education and 1 = commenced or completed vocational or higher education.

**Table 4:** Multiple regression analysis of student’s self-directed learning readiness

Variable	B	SE	β	*t*	*p*	Tolerance	VIF
(Constant)	47.550	9.391		5.063	< 0.001		
Age	0.315	0.216	0.063	1.456	0.146	0.673	1.486
Female	−0.477	2.150	−0.10	−0.222	0.825	0.690	1.449
Vocational/Higher Education	7.191	2.214	0.139	3.248	0.001	0.697	1.435
Personality factor							
Extrovert	0.144	0.166	0.036	0.866	0.387	0.731	1.368
Intellect/imagination	1.682	0.179	0.388	9.386	< 0.001	0.747	1.339
Conscientiousness	0.924	0.163	0.235	5.657	< 0.001	0.739	1.353
Agreeableness	1.132	0.189	0.251	5.988	< 0.001	0.726	1.377
Emotional Stability	0.236	0.120	0.075	1.959	0.051	0.872	1.147
Program							
Occupational Therapy	7.645	2.876	0.116	2.658	0.008	0.668	1.498
Physiotherapy	10.670	2.993	0.155	3.565	< 0.001	0.674	1.484
Podiatry	9.186	3.706	0.105	2.479	0.014	0.719	1.390
Sports and Exercise Science	2.710	2.523	0.053	1.074	0.284	0.535	1.870

A significant regression equation was found *F*(12,368) = 34.464, *p* < 0.001, with an *R* ^2^ of 0.529. The scores of students who had either commenced or completed vocational or higher education were 7.19 points higher than students who had only completed high school, controlling for age, gender, discipline and personality factors. ‘Conscientiousness’ (*p* < 0.001), ‘agreeableness’ (*p* < 0.001), and ‘intellect/imagination’ (*p* < 0.001) were each significant with each additional point in those scales associated with a respective increase of 0.92, 1.13 and 1.68 marks in the SDLRS, when holding the other factors constant. The SDLRS scores of physiotherapy students were 10.67 marks higher (*p* < 0.001), podiatry students 9.12 marks higher (*p* = 0.014), occupational therapy students 7.65 marks higher (*p* = 0.008), and sports and exercise science students 2.71 marks higher (*p* = 0.284) than students in health sciences (therapeutic recreation, health promotion and health services management). There were no significant differences between genders or with age.

## Discussion

The aim of this study was to investigate potential determinants of SDLR in first year students from a range of health disciplines who were enrolled in an interprofessional subject and to explain variability in SDLR. Previously investigated factors of age, gender, highest level of previous education and discipline were examined and a new factor of personality included. Congruent with the existing literature, SDLR increased with age and with level of previous education. With regard to previous education, there appeared to be a cumulative effect with SDLR scores increasing as the level of previous education increased from secondary education, to students who commenced but did not finish a Bachelor level program and those who completed a Bachelor’s level program.

This sample had a better gender balance than many previous studies which have been overwhelmingly female. This meant the association between gender and SDLR could be meaningfully explored. Gender was associated with SDLR; females had higher scores than males. This lends support to previous findings by Kell [4] similarly in an undergraduate program, however their study was in the United Kingdom and only examined physiotherapy students. Given the routine use of gender as a factor to characterize samples it is surprising that so little evidence is available about the influence of this factor. While this study contributes important information regarding males, further work using samples with good representation from both genders is needed.

This study was the first to investigate the influence of personality on SDLR in undergraduate health discipline students. There was a significant positive relationship between SDLR and scores on each of the ‘big five’ factors: ‘extroversion’, ‘agreeableness’, ‘conscientiousness’, ‘emotional stability’, and ‘intellect/imagination’.

There was also a relationship between disciplines and SDLR score. We observed that the highest mean SDLRS scores were achieved in the programs which were the most competitive to gain entry into. Academic entry threshold scores were between 10 and 30 points higher in occupational therapy, physiotherapy, and podiatry, compared to the other programs, and SDLR means were higher in occupational therapy, physiotherapy and podiatry. This observation is of particular interest when considered in light of personality findings that showed the strongest of all trait relationships was between ‘intellect/imagination’ and higher SDLR. If entry scores are taken as a ‘proxy’ for academic ability and/or capacity, these findings invite speculation that traits may indeed be associated with SDLR variation but this should be further investigated. This, may provide useful insights particularly as SDLR is expected for health professionals in a diverse disciplinary environment.

This study is one of a few to investigate the practical significance of findings using effect size – most studies only report statistical significance. Lakens [37] iterates that effect size is useful in communicating the practical significance of results using standardised metrics, allowing researchers to draw meta-analytic conclusions by comparing across studies, and in planning future studies, particularly with respect to power analyses and sample sizes. Using Cohen’s [38] guidelines, gender *(d* = 0.264), age (*r* = 0.266), highest previous level of education (η^2^ = 0.066) and discipline (η^2^ = 0.05) each presented only small effects. At best, age was nearing the medium effect threshold of 0.3. The implications of this result are that (a) there may be other factors not considered (for example cultural or linguistic background), or (b) it is actually the combination of these factors which is important (e.g., age could be intimately linked to the time taken to get higher qualifications, or the ability to pursue previous qualifications could indicate higher academic ability related to specific disciplines) or (c) one or more of the factors examined are proxy measures for another construct not as yet named. Each of the ‘big five’ personality traits, however, showed independent effects (ranging from small to large), with ‘intellect/imagination’ demonstrating a large effect (*r* = 0.54). From a variables point of view, this study highlights the need for age, gender and discipline to be routinely included and reported as potential influencing factors. Further it highlights the value in moving beyond ‘one-size-fits-all’ demographic indicators to variables that may tell us more about students as individual people – their personality and academic capacity or performance. This will help teachers and researchers gain a more nuanced understanding of influencing factors. Expanding the range of factors examined in SDLR research may also help inform debate regarding whether or not SDLR can be ‘taught’ and what factors moderate increases or decreases over time.

This study explains much of the observed variance in SDLR. Regression analyses revealed that personality factors (‘intellect/imagination’, ‘conscientiousness’, ‘agreeableness’ and ‘emotional stability’) together with prior post-secondary education (complete or incomplete), and discipline (occupational therapy, physiotherapy and podiatry) accounted for 52.9% of variance. Prior SDLR research has not attempted to account for variance, which makes the contribution of this adequately powered multi-factor study to a useful one to educators and health professionals alike. Study findings highlight the need for future SDLR research to explore characteristics like personality, to better understand what factors may be potent in SDLR.

The study presents information that will contribute to the debate regarding whether or not SDLR can be taught. The regression analysis provided a model where much of the variance was accounted for by traits. Firstly, four of the ‘big five’ personality factors were significant in the model. In trait theory, personality traits are considered to be relatively stable over time [39]. Thus, since personality traits were associated with varying SDLR in this study, it may be reasonable to propose that personality traits “predispose” students to higher or lower SDLR. This has implications for education; it may be that certain ‘dispositions’ provide students with habits, ways of thinking or emotions that align more or less with behaviours encapsulated in SDLR. But whether or not SDLR is a “trait” per se, as suggested by Lounsbury et al. [27], needs investigation. One approach for such investigation is to see whether SDLR changes over time, given traits are relatively stable.

Study findings, while being suggestive of the involvement of traits, also suggest that there are aspects of SDLR which are related to learning. Previous level of education was significant in the regression analysis, controlling for age, yet age alone was not significant. This indicates that there must be ‘something’ learned in previous higher education experiences which influenced SDLR, given temporal or developmental factors (as indicated by age) do not appear to have an effect. Research is needed to determine if there are generic skills or attitudes learned across higher education experiences which influence SDLR, or whether exposure to particular disciplines’ teaching and learning approaches yields greater influences. Irrespective of the degree to which SDLR can be taught, it is evident that different educational strategies may be needed in health professional training, given the diversity of students, both in personality traits and previous educational experiences.

## Limitations

This study was conducted at one metropolitan university as a single cohort cross-sectional study. There is, therefore, limited opportunity for comparison across a range of disciplines and locations. Also, given this study is the first of its kind to explain variance in SDLR of students in pre-certification health discipline programs, there is no comparative study.

## Conclusion

Congruent with the existing literature, SDLR increases with age, highest level of previous education, and most notably, with increasing scores in each of the ‘big five’ personality traits. While each of the factors investigated had a modest association with SDLR, in combination, personality traits and previous education level could account for half the variance. Further research should explore how individual student characteristics such as personality (investigated here), and other factors not explored such as cultural and linguistic diversity, socio-economic status and academic capacity, might affect SDLR. Further research should include not only self-report measures of SDLR but also in performance measures where behaviour indicative of SDLR can be observed. As workforce diversity expands and expectations for graduate SDL capacity continue, future research needs to consider all possible influences on readiness.

## Abbreviations

ANOVA: Analysis of variance
IPIP: International Personality Item Pool
SDL: Self-directed learning
SDLR: Self-directed learning readiness
SDLRS: Self-directed learning readiness scale
SDLRSNE: Self-directed learning readiness scale for nursing education

## References

[1] Knowles MS (1975). Self-directed learning: a guide for learners and teachers.

[2] Slater CE, Cusick A. Factors related to self-directed learning readiness of students in health professional programs: a scoping review. Nurse Educ Today. 2017;52:28–33. doi:10.1016/j.nedt.2017.02.011 10.1016/j.nedt.2017.02.011 28229917

[3] Harvey BJ, Rothman AI, Frecker RC (2003). Effect of an undergraduate medical curriculum on students’ self-directed learning. Acad Med.

[4] Kell C (2006). Undergraduates’ learning profile development: what is happening to the men?. Med Teach.

[5] Kell C, van Deursen R (2000). The fight against professional obsolescence should begin in the undergraduate curriculum. Med Teach.

[6] Linares AZ (1989). A comparative study of learning characteristics of RN and generic students. J Nurs Educ.

[7] Linares AZ (1999). Learning styles of students and faculty in selected health care professions. J Nurs Educ.

[8] Premkumar K, Pahwa P, Banerjee A, Baptiste K, Bhatt H, Lim HJ (2013). Does medical training promote or deter self-directed learning? A longitudinal mixed-methods study. Acad Med.

[9] Williams B (2004). Self direction in a problem based learning program. Nurse Educ Today.

[10] Duman ZC, Sen H (2012). Longitudinal investigation of nursing students’ self-directed learning readiness and locus of control levels in problem-based learning approach. New Educ Rev.

[11] Klunklin A, Viseskul N, Sripusanapan A, Turale S (2010). Readiness for self-directed learning among nursing students in Thailand. Nurs Health Sci.

[12] Kocaman G, Dicle A, Ugur A (2009). A longitudinal analysis of the self-directed learning readiness level of nursing students enrolled in a problem-based curriculum. J Nurs Educ.

[13] Phillips BN, Turnbull BJ, He FX (2015). Assessing readiness for self-directed learning within a non-traditional nursing cohort. Nurse Educ Today.

[14] O'Kell SP (1988). A study of the relationships between learning style, readiness for self-directed learning and teaching preference of learner nurses in one health district. Nurse Educ Today.

[15] Premkumar K, Pahwa P, Banerjee A, Baptiste K, Bhatt H, Lim HJ (2014). Changes in self-directed learning readiness in dental students: a mixed-methods study. J Dent Educ.

[16] Kar SS, Premarajan KC, Ramalingam A, Iswarya S, Sujiv A, Subitha L (2014). Self-directed learning readiness among fifth semester MBBS students in a teaching institution of South India. Educ Health (Abingdon).

[17] Shokar GS, Shokar NK, Romero CM, Bulik RJ (2002). Self-directed learning: looking at outcomes with medical students. Fam Med.

[18] Davis JH, Pearson MA (1996). An instructional model for primary health care education. Public Health Nurs.

[19] Gagnon M-P, Gagnon J, Desmartis M, Njoya M (2013). The impact of blended teaching on knowledge, satisfaction, and self-directed learning in nursing undergraduates: a randomized, controlled trial. Nurs Educ Perspect.

[20] Kell C, van Deursen R (2002). Student learning preferences reflect curricular change. Med Teach.

[21] Kell C, van Deursen R (2003). Does a problem-solving based curriculum develop life-long learning skills in undergraduate students?. Physiotherapy.

[22] Deyo ZM, Huynh D, Rochester C, Sturpe DA, Kiser K (2011). Readiness for self-directed learning and academic performance in an abilities laboratory course. Am J Pharm Educ.

[23] Huynh D, Haines ST, Plaza CM, Sturpe DA, Williams G, Rodriguez de Bittner MA (2009). The impact of advanced pharmacy practice experiences on students’ readiness for self-directed learning. Am J Pharm Educ.

[24] Walker JT, Lofton SP (2003). Effect of a problem based learning curriculum on students’ perceptions of self directed learning. Issues. Educ Res.

[25] Malta S, Dimeo SB, Carey PD (2010). Self-direction in learning: does it change over time?. J Allied Health.

[26] Doherty EM, Nugent E (2011). Personality factors and medical training: a review of the literature. Med Educ.

[27] Lounsbury JW, Levy JJ, Park S-H, Gibson LW, Smith R. An investigation of the construct validity of the personality trait of self-directed learning. Learn Indiv Differ. 2009;19(4):411–8. doi:10.1016/j.lindif.2009.03.001

[28] Guglielmino L (1978). Development of the self-directed learning readiness scale: University of Michigan.

[29] Goldberg LR (1999). A broad-bandwidth, public domain, personality inventory measuring the lower-level facets of several five-factor models. Pers Psychol Eur.

[30] Guglielmino, Associates L. Learning Preference Assessment. http://www.lpasdlrs.com. Accessed 4 Feb 2016.

[31] Guglielmino L, Guglielmino P (1991). Learning preference assessment facilitator guide.

[32] Wiley KR (1981). Effects of a self-directed learning project and preference for structure on the self-directed learning readiness of baccalaureate nursing students.

[33] Finestone P (1984). A construct validation of the self-directed learning readiness scale with labour education participants.

[34] Fisher M, King J, Tague G (2001). Development of a self-directed learning readiness scale for nursing education. Nurse Educ Today.

[35] Goldberg LR (1992). The development of markers for the big-five factor structure. Psychol Assess.

[36] Gow AJ, Whiteman MC, Pattie A, Deary IJ (2005). Goldberg’s ‘IPIP’Big-five factor markers: internal consistency and concurrent validation in Scotland. Pers Individ Dif.

[37] Lakens D (2013). Calculating and reporting effect sizes to facilitate cumulative science: a practical primer for t-tests and ANOVAs. Front Psychol.

[38] Cohen J (1988). Statistical power analysis for the behavioral sciences.

[39] Roberts BW, Wood D, Caspi A, John OP, Robins RW, Pervin LA (2008). The development of personality traits in adulthood. Handbook of personality: theory and research.

